# An Investigation into the Antiobesity Effects of *Morinda citrifolia* L. Leaf Extract in High Fat Diet Induced Obese Rats Using a ^1^H NMR Metabolomics Approach

**DOI:** 10.1155/2016/2391592

**Published:** 2015-12-20

**Authors:** Najla Gooda Sahib Jambocus, Nazamid Saari, Amin Ismail, Alfi Khatib, Mohamad Fawzi Mahomoodally, Azizah Abdul Hamid

**Affiliations:** ^1^Faculty of Food Science and Technology, Universiti Putra Malaysia, 43400 Serdang, Selangor, Malaysia; ^2^Faculty of Medicine and Health Sciences, Universiti Putra Malaysia, 43400 Serdang, Selangor, Malaysia; ^3^Department of Pharmaceutical Chemistry, Kulliyyah of Pharmacy, International Islamic University Malaysia, 25200 Kuantan, Pahang, Malaysia; ^4^Department of Health Sciences, Faculty of Science, University of Mauritius, 230 Réduit, Mauritius; ^5^Halal Products Research Institute, Universiti Putra Malaysia, 43400 Serdang, Selangor, Malaysia

## Abstract

The prevalence of obesity is increasing worldwide, with high fat diet (HFD) as one of the main contributing factors. Obesity increases the predisposition to other diseases such as diabetes through various metabolic pathways. Limited availability of antiobesity drugs and the popularity of complementary medicine have encouraged research in finding phytochemical strategies to this multifaceted disease. HFD induced obese Sprague-Dawley rats were treated with an extract of *Morinda citrifolia* L. leaves (MLE 60). After 9 weeks of treatment, positive effects were observed on adiposity, fecal fat content, plasma lipids, and insulin and leptin levels. The inducement of obesity and treatment with MLE 60 on metabolic alterations were then further elucidated using a ^1^H NMR based metabolomics approach. Discriminating metabolites involved were products of various metabolic pathways, including glucose metabolism and TCA cycle (lactate, 2-oxoglutarate, citrate, succinate, pyruvate, and acetate), amino acid metabolism (alanine, 2-hydroxybutyrate), choline metabolism (betaine), creatinine metabolism (creatinine), and gut microbiome metabolism (hippurate, phenylacetylglycine, dimethylamine, and trigonelline). Treatment with MLE 60 resulted in significant improvement in the metabolic perturbations caused obesity as demonstrated by the proximity of the treated group to the normal group in the OPLS-DA score plot and the change in trajectory movement of the diseased group towards the healthy group upon treatment.

## 1. Introduction

The new understanding of obesity and its related disorders has resulted in a renewed interest in finding antiobesity agents from nature, with partial success [1]. An established antiobesity agent, such as green tea polyphenols, is one of the few plants extracts reported to reduce weight in both animals and human subjects [2–4]. Others include extracts of Nomame Herba, cocoa, and chitin/chitosan [5–7]. While these studies yielded significant information on the effect of those plants on diet induced obesity and its biochemical changes, the overall effect on metabolic responses is relatively unknown.

Metabolomics as a new bioanalytical technique in obesity research is still largely unexplored. This “omics” technique is concerned with the high throughput identification and quantification of small molecules (<1500 Da) in the metabolome, the collection of small metabolites present in a cell, organ, or organism [8]. So far, the main application of the metabolomics approach has been in toxicological and pharmaceutical research, having the potential of “bridging Traditional Chinese Medicine (TCM) and molecular pharmacology” [9]. Metabolomics has been applied for extracts characterisation and quality control of herbal supplements [10]. In regard to disease biomarkers discovery, metabolomics, in combination with multivariate data analysis, has been used for the profiling of various biofluids [11, 12]. More specifically to obesity research, it has been used to discriminate between metabolites of the obese models and the healthy models [13–15]. Metabolites such as betaine, taurine, acetone/acetoacetate, phenylacetylglycine, pyruvate, lactate, and citrate were the main discriminating metabolites between the obese and lean groups [13]. There is also the emerging trend of using metabolomics as a platform to study the holistic efficacy of traditional medicine. ^1^H NMR based metabolomics approach was used to assess the effect of Xue-Fu-Zhu-Yu decoction (XFZYD) on high fat diet induced hyperlipidemia in rats. Metabolomics analysis of the plasma, combined with multivariate data analysis, revealed that XFZYD improved hyperlipidemia by regulating major metabolic pathways such as decreasing the accumulation of ketone bodies, enhancing glutathione biosynthesis, and reversing disturbances in lipid and energy metabolism [16].

*Morinda citrifolia* L., commonly called noni or Indian Mulberry, was discovered by the Polynesians more than 2000 years ago and brought to southeast Asia during migration [17]. Different parts of the plant have a long history of safe use and were reported to have many health promoting properties [18] including antidyslipidemic effects in rats [19] and inhibition of digestive and metabolic lipases* in vitro* [20–22]. We recently showed (results under publication) that a rutin rich extract of* Morinda citrifolia* leaves (MLE60) prevented weight and fat mass gain in lean Sprague-Dawley rats fed with a high fat diet with an improvement in plasma lipids, leptin, and insulin profiles and increased fecal fat output.

In this study, we assessed the effects of the leaf extract in high fat diet induced obese male Sprague-Dawley rats, using a ^1^H NMR metabolomics approach, analysing urine and serum for markers metabolites.

## 2. Methodology

### 2.1. Preparation of* Morinda citrifolia* Leaf Extract (MLE 60)

Mature* M. citrifolia* leaves were obtained from 5 representative trees from Bukit Expo, Universiti Putra Malaysia, Serdang, Selangor, Malaysia. Voucher specimens were deposited at the herbarium, Institute of Bioscience, Universiti Putra Malaysia (SK2197/13), and species were confirmed as* M. citrifolia* L. The leaves were immediately quenched using liquid nitrogen and lyophilised under pressure (−50°C, 48–72 hours, LABONCO, Labonco Corporation, Kansas City, Missouri, USA) until constant weight. The dried plant sample was ground using a commercial grinder, sieved, and stored at −80°C until further use.

Dried plant materials were extracted with 60% ethanol at room temperature for 72 hours. Filtrate was collected every 24 hours and the pooled filtrate was rotary-evaporated under vacuum until being concentrated. The aqueous phase was frozen at −80°C and lyophilised under pressure (−50°C, 48 hours) and stored at −80°C until future use. Extracts were prepared by dissolving weighed amount of extract in 0.03% carboxymethyl cellulose (CMC).

### 2.2. Animal Experiment

Male Sprague-Dawley rats (3 weeks old) were purchased from Sapphire Enterprise, Malaysia, and acclimatized for 10 days under standard laboratory conditions (12 h light/dark cycle, 55–60% relative humidity, 23–25°C). After acclimatization, rats were randomly divided into 2 groups based on assigned diets: standard rat chow (Gold Coin, Malaysia) and a high saturated fat diet for 12 weeks (MP Diets, USA). The body weight of each rat in both groups was recorded weekly to ensure development of obesity in the HFD group. After 12 weeks of the assigned diet, rats in the HFD group were then further divided into the following groups (*n* = 6), based on supplementation or nonsupplementation with MLE 60/Orlistat, and rats in both HFD and ND groups were continued on their respective diets:

(i) ND: normal diet only.
(ii) HFD: high fat diet only.
(iii) HFD + 250: high fat diet + 250 mg/kg body weight MLE 60.
(iv) HFD + 500: high fat diet + 500 mg/kg body weight MLE 60.
(v) HFD + OR: high fat diet + 30 mg/kg body weight Orlistat.

**Figure 1:**
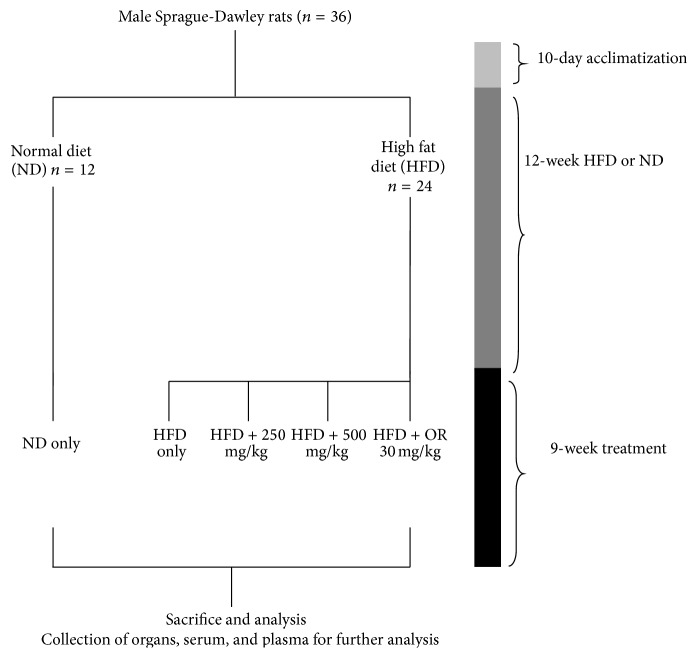
Schematic diagram of the experimental design to assess the antiobesity effect of MLE 60 in HFD induced obese male Sprague-Dawley rats.

Orlistat, the currently available pancreatic lipase inhibitor, was used as positive control. An overview of the experiment is given in Figure 1.

### 2.3. Administration of MLE 60

Animals were allowed their respective diets* ad libitum* and required dosage of MLE 60 was given through gastric intubation. Volume of extracts given per day did not exceed 3 mL. Control groups (ND and HFD) received the vehicle (0.03% CMC) through gastric intubation. Body weight and food intake of each rat were recorded weekly.

### 2.4. Urine, Serum, and Feces Collection

Animals were placed in individual metabolic cages at the initial, middle, and final stages of the experiment. Urine was collected over 24 hours in tubes containing 1% sodium azide, transferred to urine specimen bottles, and stored at −80°C until being analysed. Blood samples were collected by cardiac puncture and serum and plasma samples were separated at 1500 ×g for 15 minutes and stored at −80°C for further analysis. Feces were collected and stored in airtight containers at −80°C for further analysis.

### 2.5. Sacrifice of Animals

After 12 weeks of obesity induction and 9 weeks of treatment, animals were weighed and sacrificed by cardiac puncture under an anaesthetic effect (xylazine + ketamine). Rats were deprived of food for 12 h prior to sacrifice. Serum and plasma samples were separated at 1500 ×g for 15 minutes and stored at −80°C for further analysis. All animals were handled according to the international principles of the Use and Handling of Experimental Animals (United States National Institute of Health, 1985) and all the protocols were approved by the Animal House and Use Committee of the Faculty of Medicine and Health Sciences, Universiti Putra Malaysia (Approval number UPM/FPSK/PADS/BR.UUH/00462).

### 2.6. Clinical Chemistry Measurements

Various biochemical parameters were measured, including blood glucose (One Touch Basic glucose monitor, LifeScan), lipids profiles (Roche Diagnostics GmbH, Sandhofer Strasse, Mannheim), total cholesterol (TC), total triglycerides (TG), low density lipoprotein (LDL), high density lipoprotein (HDL), kidney function tests (creatinine and urea), liver function tests *γ*-glutamyltransferase (GGT), alanine aminotransferase (ALT), aspartate aminotransferase (AST), alkaline phosphatase (ALP), leptin (RayBio Rat Leptin ELISA kit, Cat# ELR-Leptin-001, Norcross, GA, USA), insulin (Mercodia Rat Insulin ELISA, Uppsala, Sweden), adiponectin (Assay-Max Rat Adiponectin ELISA kit, Cat# ERA2500-1), and ghrelin (RayBio Rat Ghrelin ELISA kit, Cat# EIA-GHR-1, Norcross, GS, USA). All procedures were carried out in accordance with the manufacturers' instruction.

### 2.7. Determination of Fecal Fat Content

Fecal lipid content was determined according to a modified method of Tsujita et al. [23]. Feces were collected at the initial and final stages of the experiment and stored at −80°C until further analysis. Feces (0.5 g) were soaked in 2 mL of deionized water for 24 hours at 4°C, followed by homogenisation by vortexing at high speed for 60 seconds. Lipids were extracted with 7.5 mL of methanol : chloroform (2 : 1, v : v) and shaken for 30 minutes, followed by addition of 2.5 mL of deionized water and 2.5 mL of chloroform and further shaking for 30 minutes. Mixture was then centrifuged at 2000 g for 15 min and the lipophilic layer from the extraction was collected and dried under vacuum. Total fat content was weighed using a laboratory balance.

### 2.8. ^1^H NMR Analysis of Urine and Serum

^1^H NMR analysis of urine and serum was carried out following the method of Beckonert et al. [12]. Urine samples were thawed and centrifuged at 12 000 ×g for 10 minutes. 400 *μ*L of the supernatant was mixed with 200 *μ*L phosphate buffer solution consisting of 0.1% of 3-trimethylsilyl propionic-2,2,3,3-d4 acid sodium salt (TSP) as internal standard (adjusted to pH 7.4 using NaOD) and transferred into 5 mm NMR tubes. Spectra were acquired at 27°C on a Varian Unity INOVA 500 MHz spectrometer (Varian Inc., CA), with a frequency of 499.887 MHz. Standard one-dimensional (1D) NOESY-presat pulse sequence was used for suppression of the water peak. For each sample, 64 scans were recorded with an acquisition time of 1.36 s, pulse width of 3.75 *μ*s, and relaxation delay of 1.0 s.

For serum, thawed samples were centrifuged at 12 000 ×g for 10 minutes and 200 *μ*L of the supernatant was mixed with 400 *μ*L of phosphate buffer containing 0.2% TSP and transferred into 5 mm NMR tubes. In addition to the NOESY-presat experiments, water suppressed Carr-Purcell-Meiboom-Gill (CPMG) spin-echo pulse was performed to suppress broad signals from macromolecules. The CPMG spectra were acquired with 128 transients, with an acquisition time of 1.36 s, relaxation delay of 2.0 s, and number of loops of *n* = 80.

Additional two-dimensional ^1^H-^1^H J resolved and ^1^H-^13^C HMBC analysis was performed to confirm the identity of certain metabolites.

### 2.9. NMR Spectral Data Reduction and Multivariate Data Analysis

Chenomx NMR Suite (Chenomx, Calgary, Canada) was used for metabolite identification and quantification. Nonzero filled spectra were manually phased and baseline corrected, calibrated to TSP at 0.00 ppm. Processed spectra (*δ* 0−10 ppm) were segmented (0.04 ppm) using the profiler module. Residual signals of water (*δ* 4.75–*δ* 4.85) and urea (*δ* 5.50–*δ* 6.00 ppm) were excluded from analysis. Remaining bins were normalized to the sum of spectral integrals, extracted with Microsoft Excel, and imported into Simca-P software (Umetrics, Umeå, Sweden) for multivariate data analysis.

Multivariate data analysis was performed using the mean centering with Pareto scaling. Principal component analysis (PCA) was selected as the initial clustering method. Partial Least Squares Discriminant Analysis (PLS-DA) was further performed as a supervised pattern recognition analysis, which maximizes the variation between the different groups and identifies variables responsible for the separation. Orthogonal projections to latent structures-discriminant analysis (OPLS-DA) were also performed for biomarkers analysis between the obese and lean groups and any metabolite changes associated with MLE 60 treatment [24].

### 2.10. Statistical Analysis

Data are expressed as mean ± standard deviation (SD). Difference between groups was determined by one-way analysis of variance (ANOVA, Minitab Version 14.0). Values were considered to be significantly different at the level of *p* < 0.05. For analysis of fecal fat content (week 6 and week 12) and body weight (before and after treatment), significance was further confirmed with one-sample *t*-test.

## 3. Results and Discussion

### 3.1. Induction of Obesity in Sprague-Dawley Rats Using a High Saturated Fat Diet

After 12 weeks of either the HFD or the ND, rats on the HFD had significantly higher weight gain as compared to rats on the ND. Sprague-Dawley rats on the HFD put on 157.54 ± 39.54% of their original weight whereas rats on the ND gained 93.34 ± 13.82%. Other obesity related biomarkers such as total triglycerides (TG), total cholesterol (TC), low density lipoprotein (LDL), and high density lipoprotein (HDL) levels in the plasma were also affected by the diet intervention (Table 1). Obese rats had lower HDL level (0.65 ± 0.08 mmol/L) as compared to lean rats (0.986 ± 0.16). There was no significant difference in the plasma TC and LDL content in both groups. The TG level was significantly (*p* < 0.05) elevated in the group fed the HFD (0.908 ± 0.15 mmol/L) as compared to rats fed the ND (0.432 ± 0.07). Obese rats had higher fasting glucose levels than lean rats, though still in the normal range. Other obesity related adipocytic factors such as leptin and insulin were elevated in the obese models. Kidney function tests as measured by plasma urea and creatinine levels appeared normal, with no significant difference between the groups. In terms of liver function, GGT, ALT, and ALP levels were increased in obese rats fed the HFD. Hypercaloric diets ranging from 3.7 to 5.5 kcal/g result in models of obesity, which represent the aetiology of obesity at its best and reproduce its pathophysiological characteristics [25]. The increase in weight gain is gradual as the intervention progresses. Based on the changes in lipid profiles and other plasma biochemistries, our study, which uses HFD containing 36% of total calories from coconut oil, supports the theory that coconut/lard based high fat diets do model the metabolic disorders of human obesity in rodents [26, 27]. More specifically, a hydrogenated coconut oil (HCO) based HFD has previously caused weight gain, increased liver weight, and hyperlipidaemia in rats [28]. Based on the significant increase in body weight and other biochemical parameters measured, we can conclude that high fat diet induced obesity was successfully achieved in male Sprague-Dawley rats after a feeding period of 12 weeks.

### 3.2. ^1^H NMR Spectra of Urine and Serum Metabolites of Sprague-Dawley Rats Fed HFD or ND for 12 Weeks

**Figure 2:**
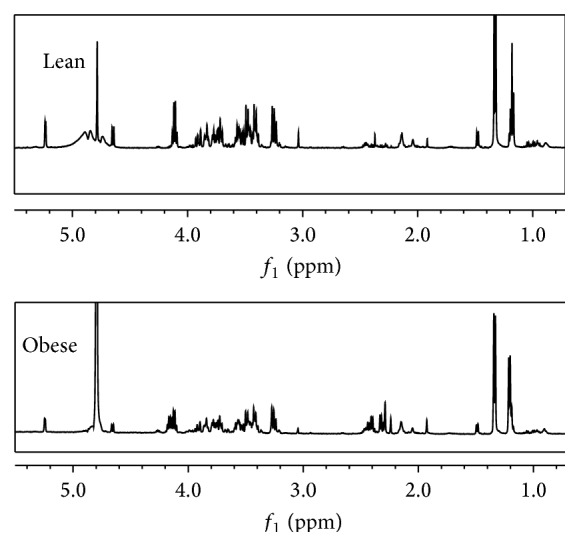
Typical 500 MHz ^1^H NMR spectra of serum collected from a Sprague-Dawley rat fed a normal diet (lean) and a Sprague-Dawley rat fed a high fat diet (obese).

**Figure 3:**
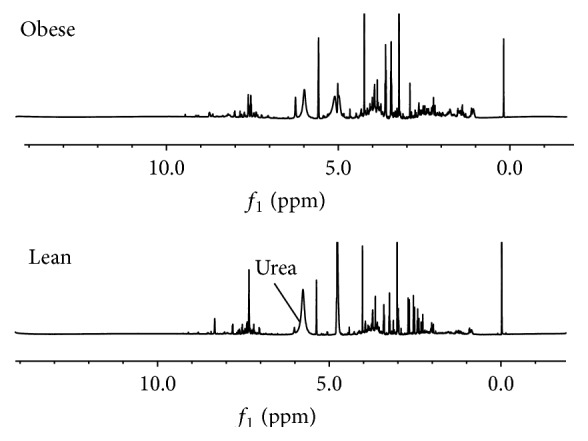
Typical 500 MHz ^1^H NMR spectra of urine collected from a Sprague-Dawley rat fed a high fat diet (obese) and a Sprague-Dawley rat fed a normal diet (lean).

Representatives of ^1^H NMR spectra for the serum and urine samples from an obese rat fed HFD and a lean rat fed ND for 12 weeks are shown in Figures 2 and 3, respectively. Expanded regions for better comparison are available in Supplementary Data sections in Supplementary Material available online at http://dx.doi.org/10.1155/2016/2391592. Metabolites were assigned based on previous studies [29, 30], the Chenomx NMR Suite, Version 7.7 (Chenomx Inc., Edmonton, AB, Canada), and the Human Urine and Serum Metabolome Databases [31, 32]. Additional two-dimensional ^1^H-^1^H J resolved and HMBC analysis was performed to aid in the identification of certain metabolites. A list of the identified metabolites, including their chemical shifts, is represented in Table 2.

CV ANOVA was used to test the significance of the models, whereby significance is achieved with a *p* value less than 0.05. Both PLS-DA and OPLS-DA models for urine and serum were validated accordingly (Table 3).

The variable importance in project (VIP) plots were generated to identify metabolites contributing significantly to the separation of the obese and the lean groups. A cut-off value of 0.7-0.8 for the VIP is generally acceptable. In this study, the cut-off value was set at 1.0 [24].

**Figure 4:**
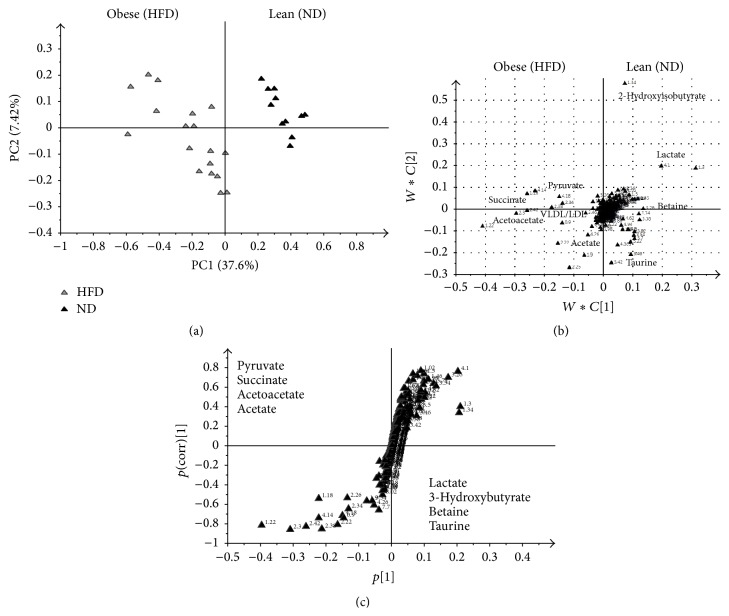
OPLS-DA derived score plot (a), loading plot (b), and *S* plot (c) obtained using ^1^H NMR data for serum samples from Sprague-Dawley rats fed a high fat diet (HFD) or a normal diet (ND) for 12 weeks.

**Figure 5:**
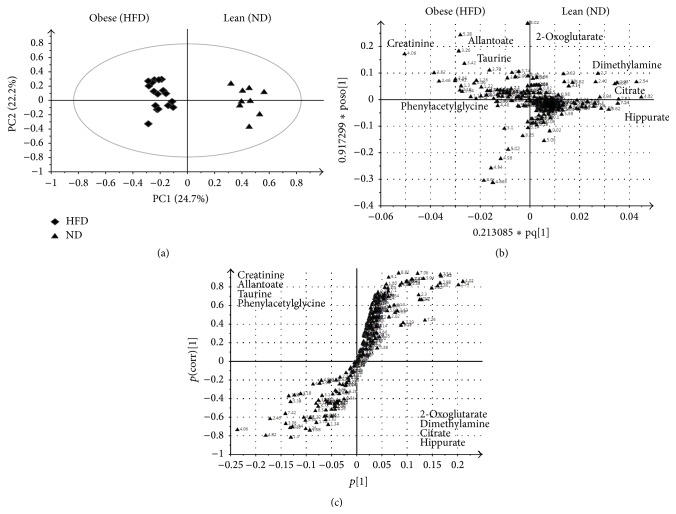
OPLS-DA derived score plot (a), loading plot (b), and *S* plot (c) obtained using ^1^H NMR data for urine samples from Sprague-Dawley rats fed a high fat diet (HFD) or a normal diet (ND) for 12 weeks.

An OPLS-DA model was used to identify discriminating metabolites between the 2 groups fed the different diets. The OPLS-DA method is useful for biomarkers identification. The *S* plot was further used to visualise the influence of the variables in the model by considering both covariance *p*(1) and correlation *p*(corr) loadings profiles from the OPLS-DA model. This enables filtering interesting metabolites in the projection. Ideal biomarkers have high magnitude and reliability values (Figures 4 and 5).

Induction of obesity was associated with increased serum levels of acetate, succinate, pyruvate, VLDL/LDL, and acetoacetate and decreased levels of lactate, 2-hydroxyisobutyrate, and betaine, among others (Figure 4). The same principle was applied to the analysis of urine and clear separation was obvious in the ^1^H NMR profiles (Figure 5). Among the increased metabolites in the HFD group were the levels of creatinine, allantoin, taurine, and phenylacetylglycine, while the levels of 2-oxoglutarate, dimethylamine, citrate, and hippurate were decreased.

After 12 weeks of feeding on the HFD, male Sprague-Dawley rats had a significantly (*p* < 0.05) higher increase in body weight as compared to rats fed ND. Obese rats had higher plasma level of TG and lower level of HDL as compared to their lean counterparts. TC and LDL levels were not significantly changed in the 2 groups fed ND and HFD. ^1^H NMR analysis of urine and serum revealed additional metabolic changes, beyond the measured small set of parameters. OPLS-DA analysis of serum ^1^H NMR spectra revealed increased succinate, pyruvate, VLDL/LDL, and acetoacetate and decreased lactate, betaine, and taurine levels in the HFD group. OPLS-DA analysis of urine samples showed that rats fed HFD had higher urinary content of creatinine, allantoin, taurine, and phenylacetylglycine and decreased levels of 2-oxoglutarate, dimethylamine, citrate, and hippurate.

All of these identified metabolites are related to various metabolic pathways, namely, the glucose metabolism and tricarboxylic acid (TCA) cycle, lipid metabolism, choline metabolism, amino acids metabolism, and creatinine metabolism.

*Glucose Metabolism and TCA Cycle*. Obese rats had higher succinate, pyruvate, acetoacetate, and acetate levels and decreased levels of lactate, 2-oxoglutarate, and citrate, all metabolites related to the glucose metabolism and the TCA cycle. These findings are consistent with other reports on the metabolomics studies of obesity [14, 33, 34]. This current study showed decreased serum lactate in obese rats fed HFD for 12 weeks, which is similar to the study of Song et al. [16] where hyperlipidemic mice fed HFD had decreased level of lactate. The urine of Zucker rats and the serum of HFD mice also had lower lactate content than the normal weight rats and the serum [14, 35]. The reduced lactate levels can be explained by factors other than HFD induced obesity, including young age and activity [36].

HFD induced obese rats had lower urinary content of 2-oxoglutarate and citrate as compared to the lean rats. Previously, Schirra et al. [37] reported a decreased level of 2-oxoglutarate in 2 mutants groups studied for altered liver metabolism. The level of citrate in the plasma is regulated by insulin, glucose levels, fatty acid utilization, and cholesterol synthesis [38] and is usually increased in HFD obese models [39] and diabetic models [40]. However, consistent with our findings, HFD induced rodents have decreased urinary citrate levels [37] which is associated with insulin resistance in humans [41]. Independent measurement of plasma insulin in this study showed a 6-fold increase in the insulin level of HFD fed obese rats (1.29 *μ*g/L) as opposed to the ND fed lean rats (0.21 *μ*g/L), which indicates that the HFD not only induced obesity in the rodents, but also caused the model to be insulin resistant, most likely caused by decreased urinary citrate excretion due to an increase in metabolic acidosis [42].

With lactate being the precursor for gluconeogenesis, any fluctuation in lactate levels indicates perturbations in glucose production and lipid synthesis in the liver [43]. The downregulation of pyruvate dehydrogenase phosphatase in obese subjects has been reported to be a defect, which signals insulin resistance [44]. Elevated concentrations of pyruvate suggest increased glycogenolysis and glycolysis to meet exceeding energy demands, similarly to the observation of serum profile of obese growing pigs [45].

*Lipid Metabolism*. The levels of betaine and taurine were changed as a result of the HFD, revealing changes in lipid metabolic pathways. Serum profiles showed lower levels of taurine, while there was an increased level of urinary taurine content. Previous studies have reported reduced taurine content in the serum, urine, and liver of various rodent models [14]. However, also in accordance with our findings, Kim et al. [13] reported increased levels of taurine in the urine of HFD fed rats. Taurine plays various biological roles in the conjugation of cholesterol, antioxidation of bile acids, osmoregulation, and calcium signalling pathways [46, 47]. The supplementation of taurine showed amelioration in obesity most likely mediated by the ability of taurine to increase fatty acid oxidation [48]. This study shows decreased taurine in the obese group, suggesting decreased fatty acids oxidation and inhibition of taurine biosynthetic enzymes related to obesity, as observed by increased levels of LDL/VLDL shown in both serum spectra and actual measured values.

*Choline Metabolism*. Pertaining to choline metabolism, the level of betaine was decreased in the HFD group, similarly to most metabolomics based obesity studies reporting decreased hepatic and urinary betaine content in HFD fed rodents [33, 34] and decreased hippurate in the serum of HFD mice [38]. In humans, lowered betaine levels are associated with obesity related disorders such as metabolic disorders, lipid disorders, and type 2 diabetes [49]. Supplementation of betaine causes increase in metabolites in the carnitine biosynthesis pathway, reduced accumulation of triglycerides in the liver, with no effect on body weight gain and increase in adipose tissue mass [50].

*Creatinine Metabolism*. Feeding of HFD diet for 12 weeks resulted in increased creatinine levels in the urine samples, in line with other reports [37, 51, 52].

*Amino Acids Metabolism*. High level of serum acetoacetate might be the indication of depletion in leucine level, an amino acid involved in insulin signalling, protein synthesis of muscle mass, and production of alanine and glutamine [53]. Alanine peaks were more prominent in the serum spectra of the lean rats as compared to obese subjects.

*Gut Microbiome Metabolism*. Changes in specific metabolites support the idea that there is a link between obesity and the gut microbiome. HFD induced obese rats showed high urinary content of phenylacetylglycine and decreased levels of hippurate and dimethylamines, metabolites involved in the gut microbiome metabolism. Hippurate is produced in the gut by microorganisms using glycine and benzoic acid as building blocks [54]. Increased hippurate level in the urine has been associated with leanness [34] and this study confirms the findings from other studies [38] that HFD induced obesity is associated with decreased urinary level of hippurate in rodent models of obesity. Increased levels of phenylacetylglycine in Sprague-Dawley rats fed HFD have been previously reported [13]. High gainers fed HFD were associated with increased levels of phenylacetylglycine as compared to low gainers on ND, which indicates an increase in the precursors produced by gut microorganisms [55]. Moreover, reduced dimethylamine levels in the obese group reflect changes in the gut microbiome derived metabolism, similarly to what is observed in leptin-deficient ob/ob mice [56]. Trigonelline was also identified in the urine samples of lean rats. It is an indicator of niacin metabolism, an essential vitamin needed as coenzyme in carbohydrate and lipid metabolism. The body's requirements for niacin can be met by dietary intake or endogenous biosynthesis through tryptophan-mediated metabolism carried out by the liver and the gut microorganisms [57]. Obesity related stress causes depletion in the glutathione stores and the decrease of trigonelline is related to depletion of *S*-adenosylmethionine, used to make up the energy stores [58]. A strong link between human gut microbiome and obesity was established with decreased urinary excretion of hippurate, trigonelline, and xanthine and increased urinary excretion of 2-hydroxybutyrate and bariatric surgery induced weight loss resulted in the loss of typical obese metabotype [59].

### 3.3. Effect of 9-Week Treatment with 250/500 mg/kg of MLE in the Obese Rats Models

After 12 weeks of inducing obesity, rats on the HFD were further divided and received either MLE 60 (250 and 500 mg/kg), Orlistat (30 mg/kg), or the carrier vehicle (CMC). Obese rats were kept on the HFD while lean rats were continued on the ND. Body weight and food intake were recorded weekly and the plasma biochemistry was analysed at the end of the experiment (Table 4).

Although there was no significant weight loss in the obese group, receiving MLE 60 or Orlistat, further weight gain was prevented in the HFD + 500 and HFD + OR group. Treatment resulted in reduced visceral fat, with the HFD group having the highest amount (6.62 ± 1.54%). The treated groups had a reduced % of visceral fat ranging from 3.34 ± 0.99 for the HFD + OR group to 4.87 ± 0.96% for the HFD + 500 group. There was no significant difference in decrease of visceral fat between the obese rats receiving 500 mg/kg MLE 60 and rats receiving standard antiobesity drug, Orlistat. No significant difference was recorded in the daily food intake among all groups. At baseline (before treatment), there was no significant difference in the fecal fat excretion in the lean and obese rats. Treatment with 500 mg/kg MLE increased the fecal fat excretion (12.64 ± 1.73%), with a comparable effect to treatment with Orlistat (15.89 ± 1.62%). The fecal fat content in the control group (HFD only) remained unchanged after 9 weeks (7.23 ± 1.01%).

Few plasma parameters were measured after 9 weeks of treatment (Table 4). With regard to lipid profiles, treatment with both 500 mg MLE 60/kg and Orlistat improved the plasma LDL level, reducing its levels to the LDL profile of lean rats. The treatment, however, failed to improve HDL levels, with the HFD + OR group having the lowest plasma HDL content of 0.56 ± 0.01 mmol/L. Lean rats on the ND have the highest level of HDL, 1.02 ± 0.09 mmol/L. The most marked effect was in the TG content, whereby HFD + 500 (0.50 ± 0.11) significantly improved the plasma TG level as compared to rats receiving the HFD only (0.93 ± 0.12).

The plasma insulin level was significantly improved in the HFD + 500 group (0.37 ± 0.13), which was similar to the ND group (0.31 ± 0.02). Similarly, plasma leptin levels were significantly improved in both HFD + 500 group (1050 ± 229 pg/mL) and HFD + OR group (1263 ± 30.10 pg/mL) as compared with the HFD group (2119 ± 176 pg/mL). Ghrelin levels were improved in all treated groups, with 250 mg/kg dosage being more potent, restoring the ghrelin levels to 54.57 ng/mL, not significantly different from the lean group (53.01 ng/mL). Adiponectin levels were not significantly different in the lean groups and the treated groups (9.25–9.87 ng/mL), except in the group treated with Orlistat (8.25 ng/mL), where the adiponectin level was not significantly different from the obese group (8.61 ng/mL). Treatment with 30 mg/kg Orlistat and 500 mg/kg MLE 60 had the most significant improvement.

In the previous section, rats fed HFD were associated with higher acetate and pyruvate and surprisingly lower lactate levels as opposed to lean rats fed ND. After an additional 9 weeks, rats fed HFD were still associated with higher acetate and pyruvate and also higher lactate level. Lactate is one of the key metabolites related to glucose metabolism and the TCA cycle, which has been reported to be higher in obese humans [60]. Increased lactate concentration has been attributed to the upregulation in anaerobic glycolysis in obese subjects and the balance between lactate production and lactate removal [14, 61]. The adipose tissue is one of the sites of lactate production, together with the skeletal muscles, erythrocytes, and brain [61, 62]. Increased lactate production can also reflect perturbations in glucose and lipid production in the liver due to the involvement of lactate as a precursor in gluconeogenesis [43] with increased serum lactate being associated with increased risk of mortality [63]. In this study, increased serum lactate in the HFD group can be attributed to the higher percentage of body fat as compared to the lean rats. Lactate levels in obese subjects are also highly dependent on insulin resistance [64]. In a study by Chen et al., normal weight subjects with normal blood glucose had the lowest plasma lactate levels, obese subjects with normal blood glucose had intermediate plasma lactate levels, and obese subjects with impaired blood glucose had the highest lactate levels. Consistent with these reports, this study shows that, after 21 weeks of feeding HFD, rats had higher insulin levels (1.83 *μ*g/L) as compared to after 12 weeks of feeding (1.30 *μ*g/L), which explains the elevated lactate levels in the obese groups. Treated groups had significant decrease in % of body fat and plasma insulin levels, which contributes to the decreased lactate plasma content [65].

Another metabolite, which was found to be strongly associated with obesity, is 2-hydroxyisobutyrate. It is involved in the gut microbiome metabolism and has been reported to be altered in leptin-deficient ob/ob mice [56] and increased in obese patients [66].

Regarding amino acid metabolism, serum alanine was increased in HFD group as compared to the lean group, in line with previous studies reporting increased alanine in the serum and liver of HFD induced mice and rats [39].

Obesity was characterised by decreased levels of 3-hydroxybutyrate, a metabolite of amino acid metabolism, which is associated with leanness and weight loss, where obese patients expressed the highest 3-hydroxybutyrate levels following bariatric surgery [66]. Early studies have also reported on the link between obesity and 3-hydroxybutyrate. Administration of the compound in obese subjects on low energy diets resulted in improved fat : lean ratio while not affecting weight loss [67]. The roles of acetoacetate and 3-hydroxybutyrate were further studied in obese and insulin dependent diabetic humans using a kinetic approach, to investigate ketone body metabolism. Obese subjects had lower ketone body de novo synthesis, with no significant clearance of 3-hydroxybutyrate from the normal healthy subjects, with 3-hydroxybutyrate being an important determinant in diabetic ketoacidosis [68]. Moreover, 3-hydroxybutyrate has also been associated with reduced food intake in obese subjects [69] and involved in the short-term and long-term effects of high fat diet in mice [14]. Won et al. also reported the downregulation of 2-hydroxybutyrate in both male and female leptin-deficient ob/ob mice [56].

In the metabolites identification, OPLS-DA model consisting of 2 groups at a time was employed, followed by the Shared and Unique Structure (SUS) plots, to compare biomarkers from 2 models.

Key discriminating metabolites as potential biomarkers in rat serum based on ^1^H NMR loading plots in the HFD, HFD + 500, and ND groups were quantified, relative to the TSP in the serum samples. Statistical analysis (Minitab Version 14) was further employed to detect significance. Focus was placed on metabolites with a VIP value of >1, as metabolites contributing more to the clustering of the different groups (Table 5).

There are limited studies, which have used a metabolomics approach to identify metabolic changes following intervention with drugs and therapeutics, including the phytochemical strategies for obesity, though the potential is vast [70]. However, there are few metabolomics based reports on weight loss as a result of weight loss intervention, including exercise and surgery. An energy-restricted diet for 8 weeks resulted in an improvement in glucose and lipid metabolism in overweight obese adults. Saturated fatty acids such as palmitic acid and stearic acid were significantly decreased as well as branched amino acid, isoleucine [71]. A lifestyle intervention in obese children, “Obeldicks,” resulted in significant weight loss and abdominal obesity, modulated by the role of phosphatidylcholine metabolism. This particular study also highlights the large interindividual variation to lifestyle intervention and the possible need of a more individualised approach to lifestyle interventions [72]. ^1^H NMR analysis also showed that while exercise can improve the metabolic disruptions associated with diet induced obesity, the effect cannot be cancelled out and diet predicts obesity better with a stronger influence on metabolites' profiles than exercise alone [14].

One of the few studies reporting the response of natural therapeutic agents in obese subjects assessed the effect of sea buckhorn and bilberry on serum metabolites in overweight women. No significant changes were observed in individual metabolites, though improvements in serum lipids and lipoproteins were observed [73]. The treatment of high fat diet induced hyperlipidemia with Xue-Fu-Zhu-Yu decoction was studied using a NMR based metabolomics approach. OPLS-DA analysis revealed the beneficial effects of the decoction, mainly through decrease in ketone bodies production, enhancement of biosynthesis, and modulation of lipid metabolism [16]. Dietary intervention of black soybean peptides in overweight human showed an increase in betaine, benzoic acid, pyroglutamic acid, and pipecolic acid, among others. VIP analysis showed L-proline, betaine, and lyso-PCs to be more correlated to the discrimination before and after treatment [74].

Treatment with MLE 60 at 250 mg/kg body weight improved serum levels of lactate, alanine, pyruvate, creatinine, and *α*-glucose, bringing their levels closer to the normal control whereas the level of 3-hydroxyisobutyrate, 3-hydroxybutyrate, and acetate remained unchanged. Similar improvements were achieved in the groups receiving 30 mg/kg body weight of Orlistat. The relative concentration of *α*-glucose was found to be most reduced, consistent with the actual biochemical measurement done previously where the Orlistat treated group had significantly lower plasma glucose (4.97 mmol/L) as compared to the lean group (6.02 mmol/L). This is consistent with the literature reporting that Orlistat in a weight loss regimen can significantly improve glucose tolerance and slows down the progression of type 2 diabetes and impaired glucose tolerance in clinical cases of obesity [75, 76].

Based on the relative quantification of certain metabolites (lactate, pyruvate, and glucose) in the treated groups, it is apparent that treatment with MLE 60 improved perturbations in various metabolic pathways, predominantly in the glucose and TCA cycle as reflected by positive modulations in lactate, pyruvate, and glucose levels. Disruptions in the creatinine and amino acid metabolic pathways were also improved as indicated by a reduction of creatinine and alanine accumulation in the obese groups treated with MLE 60. The levels of 3-hydroxyisobutyrate, a metabolite of the gut microbiome metabolism, were unchanged in the treated groups, suggesting that MLE 60 did not impact on the obesity-induced disruptions in the gut microbiome. Similarly, the levels of 2-hydroxybutyrate, a metabolite of amino acid metabolism, were also unchanged.

Obesity has been characterised by an elevated TCA function in diet induced hepatic insulin and fatty liver as well as decreased brain glucose metabolism, predominantly through the TCA cycle [77, 78]. Treatment with MLE 60 improved serum creatinine profiles as shown by ^1^H NMR measurement as compared to nonsignificance observed when blood creatinine level was measured. Poor creatinine clearance is associated with weight gain and central obesity due to increased metabolic abnormalities as risk factors [79]. An increase in creatine kinase and adenylate kinase 1 activity was observed in obese subjects, attributed to a compensatory effect of the downregulation of muscle mitochondrial function, associated with obesity [80].

Hence, antiobesity agent which can positively influence these pathways as well as other parameters such as adipocytes factors and weight loss shows promise for weight management.

## 4. Conclusion

Based on the reported health properties of* M. citrifolia*, including the antiobesity activities, the aim of this study was to further explore the effect of a leaf extract, MLE 60, on obesity using a ^1^H NMR metabolomic approach. ^1^H NMR spectroscopy and multivariate data analysis revealed clear metabolic differences in the urine and serum samples of the HFD induced obese and lean rats. An OPLS-DA method was chosen to project maximum separation between the groups and to identify discriminating biomarkers. All multivariate models including PLS-DA and OPLS-DA were duly validated, using permutation tests, *R* ^2^ *Y*, *Q* ^2^ *Y*, and *p* CV ANOVA values. Several metabolites were identified in both the serum and urine samples, which were the basis of difference among the groups. These metabolites were involved in the glucose metabolism and TCA cycle (lactate, 2-oxoglutarate, citrate, succinate, pyruvate, and acetate), amino acid metabolism (alanine, 2-hydroxybutyrate), choline metabolism (betaine), creatinine metabolism (creatinine), and gut microbiome metabolism (hippurate, phenylacetylglycine, dimethylamine, and trigonelline). Some key metabolites were identified and quantified showing a statistically (*p* < 0.05) significant improvement in this treated group (500 mg/kg). This study, therefore, confirms the metabolic alteration caused by HFD induced obesity in a rat model and the improvement in certain metabolic pathways, upon treatment with MLE 60. It also provides additional information that ^1^H NMR metabolomics can be a good approach to study the development of disease and response to treatment in obese subjects.

## Supplementary Material

Expanded regions of 500 MHz ^1^HNMR spectra for serum and urine collected from lean Sprague- Dawley rat fed a Normal Diet (ND) or obese Sprague- Dawley rat fed a coconut oil based high fat diet (HFD) to allow better comparison in metabolic profiles of the 2 groups.

## Figures and Tables

**Table 1:** The plasma biochemistry of rats fed a normal diet (ND) or a high fat diet (HFD) for 12 weeks to induce obesity.

	ND	HFD
Total cholesterol (mmol/L)	1.28 ± 0.15^a^	1.16 ± 0.09^a^
HDL (mmol/L)	0.99 ± 0.16^a^	0.66 ± 0.08^b^
LDL (mmol/L)	0.28 ± 0.02^a^	0.23 ± 0.03^a^
Triglycerides (mmol/L)	0.43 ± 0.07^a^	0.91 ± 0.15^b^
Leptin (pg/mL)	719.30 ± 150.1^a^	1819.50 ± 150.1^b^
Insulin (*μ*g/L)	0.20 ± 0.02^a^	1.30 ± 0.09^b^
Adiponectin (ng/mL)	7.40 ± 0.50^a^	6.11 ± 0.07^b^
Glucose (mmol/L)	5.68 ± 0.33^a^	6.26 ± 0.13^b^
Urea (*μ*mol/L)	6.26 ± 0.81^a^	5.14 ± 0.80^a^
Creatinine (mmol/L)	55.20 ± 2.17^a^	51.60 ± 2.41^a^
GGT (U/L)	1.00 ± 0.00^a^	6.00 ± 0.84^b^
AST (U/L)	76.36 ± 3.16^a^	75.40 ± 1.29^a^
ALT (U/L)	37.64 ± 5.26^a^	30.82 ± 1.48^b^
ALP (U/L)	69.14 ± 9.98^a^	127.20 ± 5.07^b^

Different small letters indicate significant difference (*p* < 0.05) between ND and HFD groups as shown by analysis of variance (ANOVA) using Minitab Version 14.

**Table 2:** ^ 1^H NMR assignments of metabolites in rat's serum and urine.

Metabolites	Assignments	Chemical shifts	Samples
Urea	NH_2_	5.78 (s)	U

Phenylacetylglycine	2,6-CH	7.42 (m)	U
	3,5-CH,	7.57 (m)	
	7-CH	7.65 (m)	
	10-CH	7.84 (m)	

Trigonelline	*γ*CH_3_	4.43 (s)	U
	C_2_H	8.1 (m)	
	C_4_H	8.8 (m)	
	C_5_H,	9.1 (s)	

Hippurate	CH_2_,	3.98 (d)	U
	CH	7.54 (d)	
	CH	7.65 (t)	

Acetate	CH_3_	1.93 (s)	U, S

Dimethylamine	CH_3_	2.71 (s)	U

Citrate	1/2CH_2_	2.54 (d)	U
	1/2CH_2_	2.66 (d)	

2-Oxoglutarate	CH_2_	2.45 (t)	U
	CH_3_	3.02 (t)	

Creatinine	CH_3_,	3.06 (s)	U
	CH_2_	4.06 (s)	

Lactate	CH_3_,	1.34 (d)	U, S
	CH	4.11 (dd)	

Β-Glucose	1-CH	4.66 (d)	U, S

*α*-Glucose	1-CH	5.22 (d)	U, S

Allantoin	CH	5.38 (s)	U

Glycine	CH_2_	3.57 (s)	U

Taurine	CH_2_S,	3.26 (t)	U, S
	CH_2_-N	3.40 (t)	

TMAO	N(CH_3_)_3_	3.26 (s)	U

Alanine	*β*CH_3_,	3.78 (dd)	S
	*α*CH	1.48 (d)	

Pyruvate	*β*CH_3_	2.38 (s)	S

Succinate	CH	2.41 (s)	S

Acetoacetate	CH_3_	2.27 (s)	S

3-Hydroxybutyrate	*γ*CH_3_	1.18 (d)	S
	*β*CH	4.23 (m)	
	*α*CH_2_	2.31 (d)	
	*α*CH_2_	2.38 (dd)	

2-Hydroxyisobutyrate	CH_3_	1.34 (s)	S

Lipoprotein	CH_3_(CH_2_)_*n*_	0.89 (m)	S

LDL/VLDL	CH_3_CH_2_CH_2_C=	1.2–1.30 (m)	S

s: singlet; d: doublet; t: triplet; dd: doublet of doublets; m: multiplet.

S: serum; U: urine.

**Table 3:** PLSDA and OPLS-DA models validation for serum and urine of Sprague-Dawley rats fed a high fat diet (HFD) or a normal diet (ND) for 12 weeks.

Samples/models	*R* ^2^ *Y*	*Q* ^2^ *Y*	*p* CV ANOVA	Number of components
Serum				
PLS-DA	0.830	0.752	3.32 × 10^−6^	2
OPLS-DA	0.987	0.936	4.26 × 10^−7^	2
Urine				
PLS-DA	0.917	0.874	3.18 × 10^−12^	3
OPLS-DA	0.959	0.923	1.89 × 10^−12^	3

**Table 4:** The body weight, % visceral fat, food intake, % fecal fat excretion, and plasma biochemistry of HFD induced obese rats after 9 weeks of treatment with MLE 60 at 250 mg/kg and 500 mg/kg body and 30 mg Orlistat/kg body weight.

	HFD	HFD + 250	HFD + 500	HFD + OR	ND
Body weight (g)					

Initial (week 12)	559.20 ± 25.89^Bb^	546.85 ± 83.20^Bb^	544.29 ± 78.74^Bb^	537.25 ± 93.83^Bb^	379.33 ± 34.82^Aa^

Final (week 21)	614.20 ± 131.58^Bb^	605.57 ± 101.50^Bb^	565.85 ± 87.47^Bb^	553.13 ± 98.93^Bb^	417.16 ± 32.99^Aa^

Visceral fat (%)	6.62 ± 1.54^c^	5.18 ± 0.40^bc^	4.87 ± 0.963^b^	3.34 ± 0.99^b^	1.70 ± 0.28^a^

Food intake (g/rat/day)	20.00 ± 3.09^a^	19.08 ± 2.29^a^	19.38 ± 2.01^a^	19.08 ± 0.86^a^	20.17 ± 1.35^a^

Fecal fat content (%)					
Initial	6.18 ± 1.19^aA^	7.35 ± 1.14^aA^	6.31 ± 1.40^aA^	6.12 ± 1.52^aA^	7.64 ± 0.70^aA^
Final	7.23 ± 1.01^aC^	9.44 ± 1.07^aC^	12.64 ± 1.73^bB^	15.89 ± 1.62^bA^	8.99 ± 0.61^aC^

Total cholesterol (mmol/L)	1.43 ± 0.08^b^	1.04 ± 0.01^a^	0.94 ± 0.02^a^	0.92 ± 0.07^a^	1.29 ± 0.13^b^

HDL (mmol/L)	0.82 ± 0.06^b^	0.57 ± 0.12^bc^	0.69 ± 0.07^b^	0.56 ± 0.01^c^	1.02 ± 0.09^a^

LDL (mmol/L)	0.33 ± 0.07^b^	0.22 ± 0.03^ab^	0.17 ± 0.03^a^	0.20 ± 0.04^a^	0.21 ± 0.05^a^

Triglycerides (mmol/L)	0.93 ± 0.16^c^	0.72 ± 0.12^bc^	0.50 ± 0.11^ab^	0.58 ± 0.01^ab^	0.42 ± 0.09^a^

Leptin (pg/mL)	2119.50 ± 176.3^b^	1563.30 ± 556.9^ab^	1050.00 ± 229.3^a^	1263.30 ± 30.10^a^	1125.00 ± 117.60^a^

Insulin (*μ*g/L)	1.83 ± 0.10^c^	0.71 ± 0.01^b^	0.37 ± 0.13^a^	0.47 ± 0.22^ab^	0.31 ± 0.01^a^

Ghrelin (ng/mL)	25.7 ± 3.71^c^	54.57 ± 4.19^a^	35.74 ± 1.68^b^	37.63 ± 0.98^b^	53.01 ± 1.95^a^

Adiponectin (ng/mL)	8.61 ± 0.77^b^	9.50 ± 0.23^ab^	9.25 ± 0.50^ab^	8.25 ± 0.44^b^	9.87 ± 0.20^a^

Glucose (mmol/L)	7.70 ± 0.78^c^	6.85 ± 0.71^bc^	5.83 ± 0.53^ab^	4.98 ± 0.17^a^	6.03 ± 0.17^b^


Different small letters indicate significant difference (*p* < 0.05) between different groups and different capital letters indicate significant difference among the same group at different time points, as shown by analysis of variance (ANOVA) using Minitab Version 14.

**Table 5:** Relative quantification of significant discriminating metabolites based on the concentration of 0.1% of 3-trimethylsilyl propionic-2,2,3,3-d4 acid sodium salt (TSP) as internal standard and quantified using Chenomx NMR Suite.

Metabolites	Chemical shifts	VIP value	HFD	ND	HFD + 250	HFD + OR	*p *value
Lactate	1.34 (d)	2.46	1662.9 ± 51.9^c^	493.4 ± 73.1^a^	482.1 ± 55.9^a^	761.0 ± 33.6^b^	0.000
	4.11 (dd)						

Alanine	3.78 (dd)	1.63	94.0 ± 3.03^b^	53.3 ± 13.48^a^	55.6 ± 3.52^a^	47.0 ± 2.50^a^	0.002
	1.48 (d)						

3-Hydroxybutyrate	1.18 (d)	3.48	316.8 ± 23.17^b^	513.8 ± 74.20^a^	368.7 ± 24.00^ab^	396.9 ± 73.54^ab^	0.023
	4.23 (m)						
	2.31 (d)						
	2.38 (dd)						

2-Hydroxyisobutyrate	1.34 (s)	5.60	232.5 ± 25.36^b^	153.8 ± 15.12^a^	143.9 ± 21.54^a^	182.1 ± 49.43^ab^	0.036

Pyruvate	2.38 (s)	2.19	55.6 ± 3.62^c^	18.7 ± 2.16^a^	31.1 ± 1.03^b^	28.3 ± 7.21^b^	0.000

Creatinine/creatine	3.06 (s)	1.28	45.1 ± 2.43^b^	25.6 ± 3.64^a^	23.3 ± 0.60^a^	39.5 ± 0.04^b^	0.000
	4.06 (s)						

*α*-Glucose	5.22 (d)	1.09	1116.4 ± 27.4^c^	458.1 ± 27.6^b^	511.7 ± 20.6^b^	206.4 ± 44.3^a^	0.000

Acetate	1.93 (s)	1.28	38.9 ± 2.80^b^	26.8 ± 4.42^a^	35.4 ± 1.50^b^	38.9 ± 2.61^b^	0.013

Different small letters indicate significant difference (*p* < 0.05) between different groups as shown by the analysis of variance (ANOVA) using Minitab Version 14.
