# Supplementary material for: An Investigation into the Antiobesity Effects of Morinda citrifolia L. Leaf Extract in High Fat Diet Induced Obese Rats Using a 1H NMR Metabolomics Approach
Source: J Diabetes Res. 2015 Dec 20;2016:2391592. doi: 10.1155/2016/2391592 (PMC4698747; doi:10.1155/2016/2391592)
Supplement: Supplementary file 1 — Expanded regions of 500 MHz 1HNMR spectra for serum and urine collected from lean Sprague- Dawley rat fed a Normal Diet (ND) or obese Sprague- Dawley rat fed a coconut oil based high fat diet (HFD) to allow better comparison in metabolic profiles of the 2 groups. [file 2391592.f1.pdf]

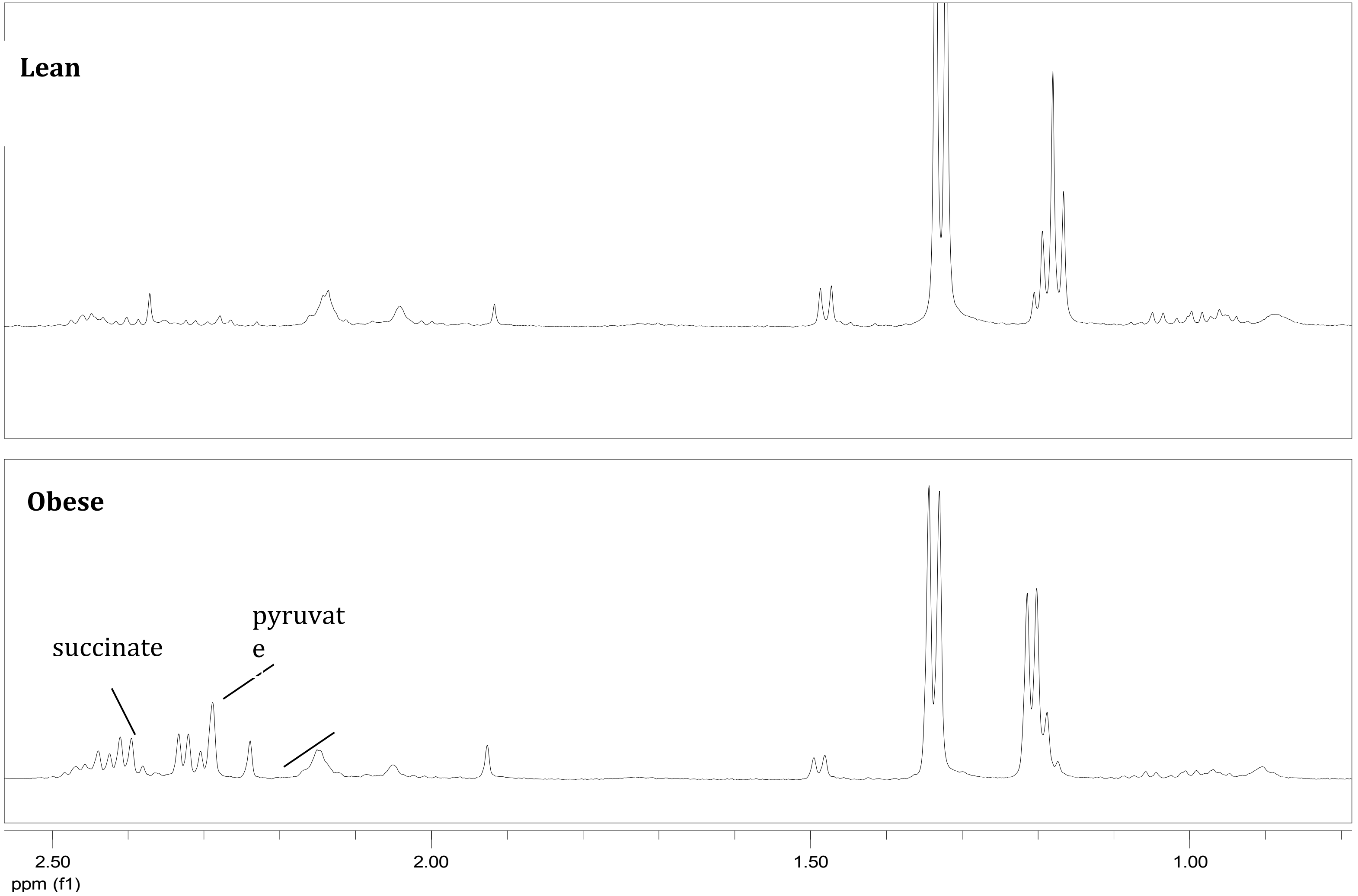
Lean
Obese
succinate
pyruvat
e
2.50
ppm (f1)
2.00
1.50
1.00

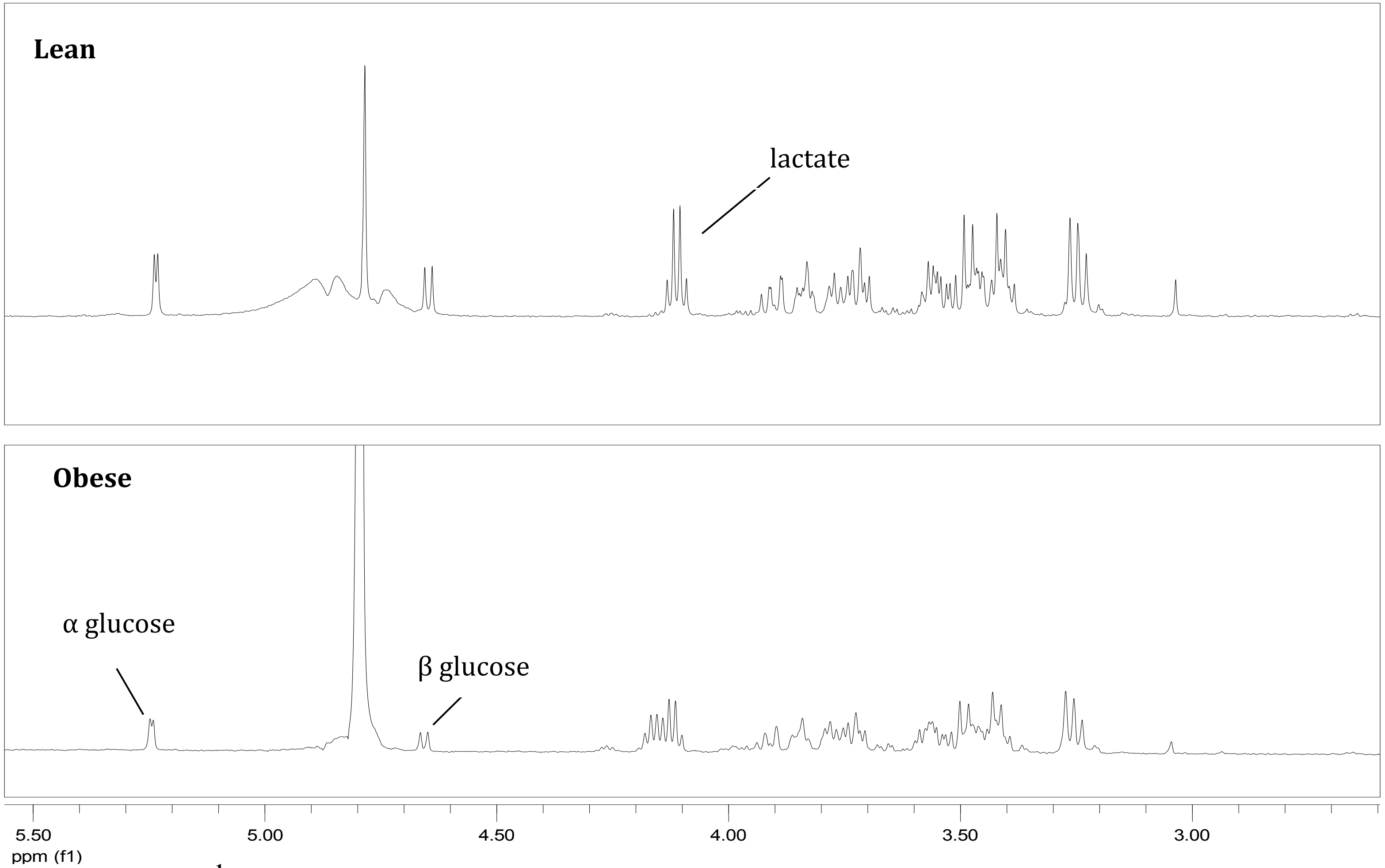

Typical 500 MHz ^{1}H NMR spectra of serum collected from a Sprague- Dawley rat fed a normal diet (lean) and aSprague- Dawley rat fed a high fat diet (obese) with expanded regions at δ 1.00 – 2.50 and δ 3.00 – 5.50 ppm.

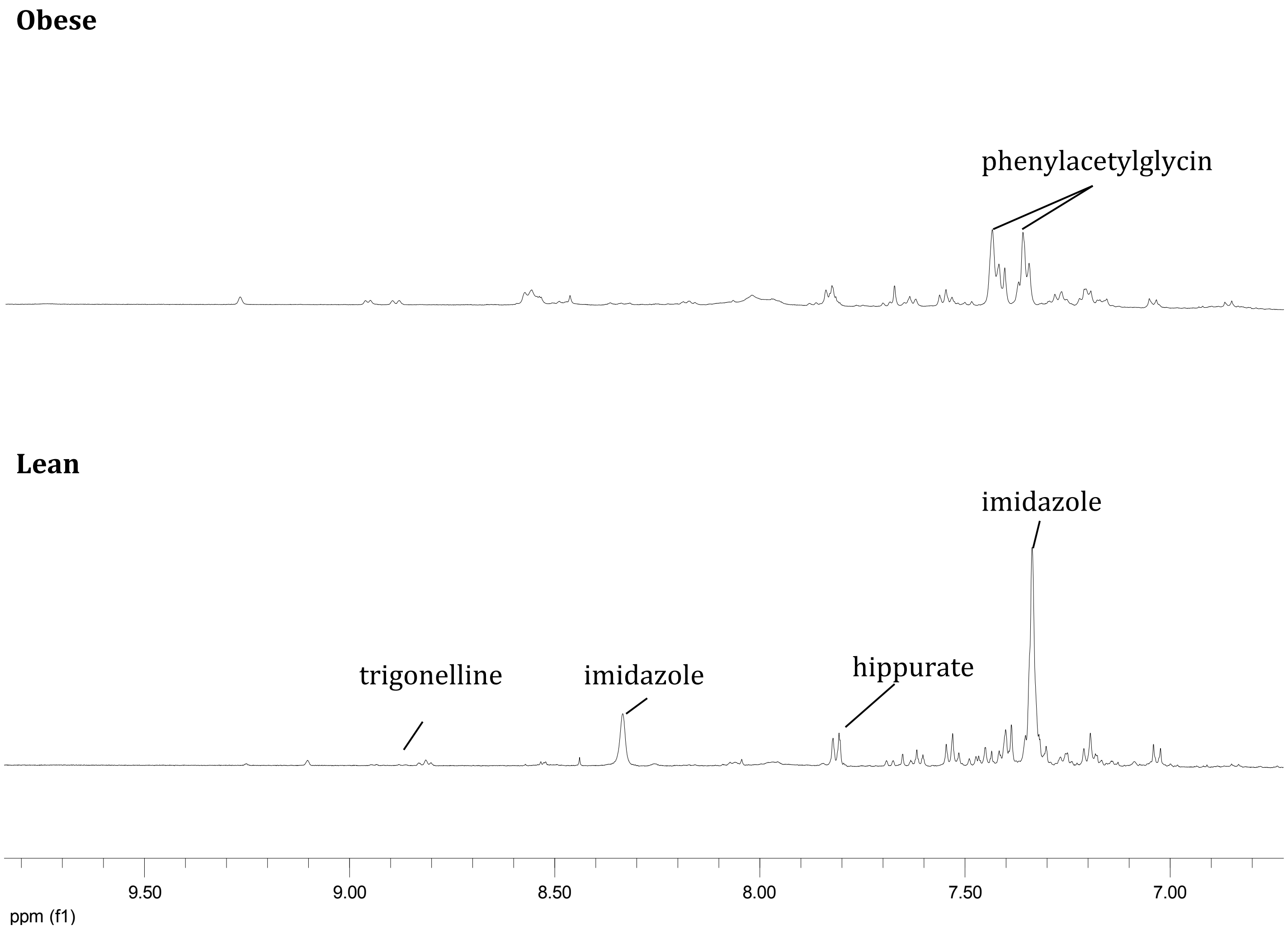
Obese
phenylacetylglycin
Lean
imidazole
trigonelline
imidazole
hippurate
9.50
9.00
8.50
8.00
7.50
7.00
ppm (f1)

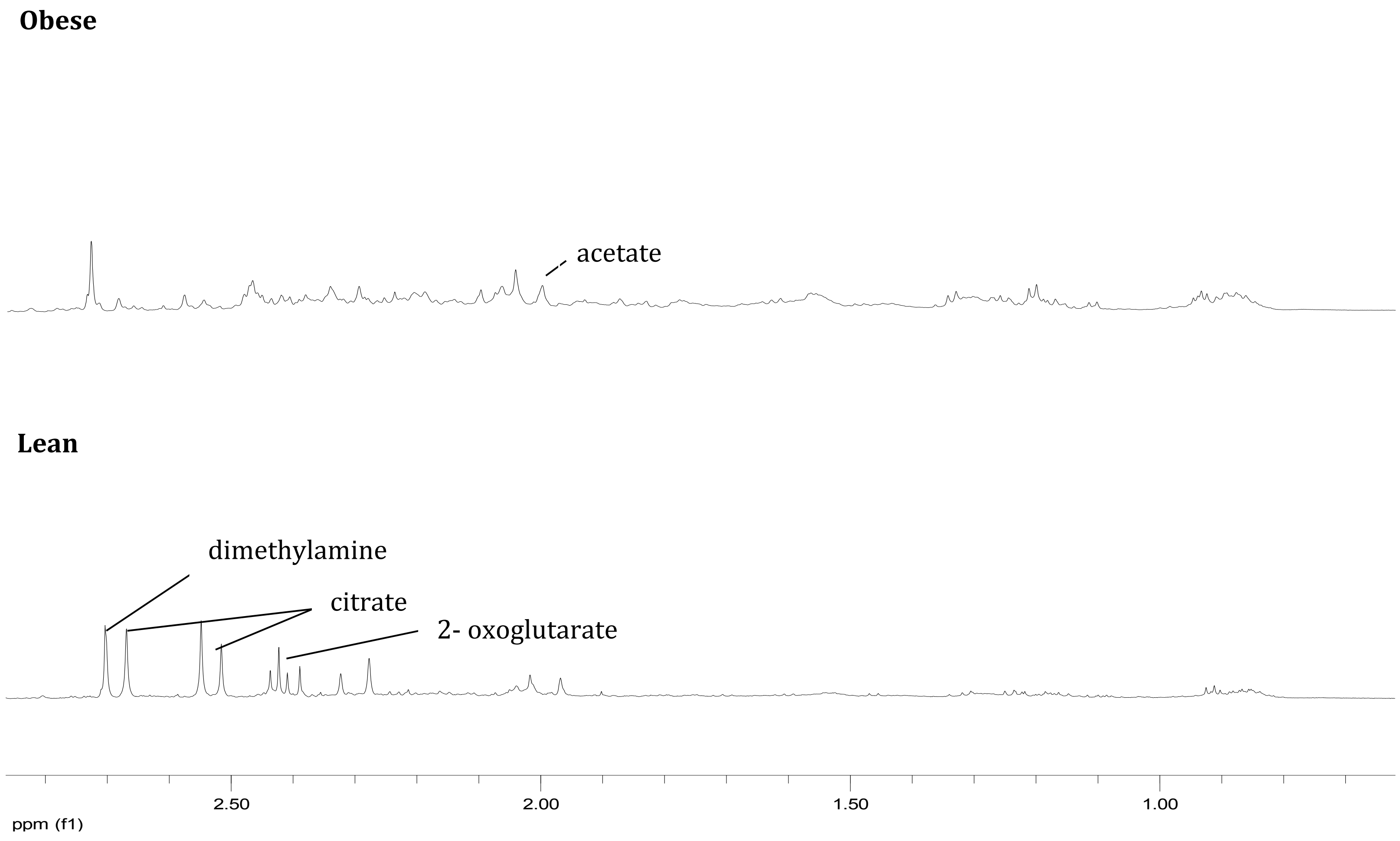
Obese
acetate
Lean
dimethylamine
citrate
2- oxoglutarate
2.50
2.00
1.50
1.00
ppm (f1)

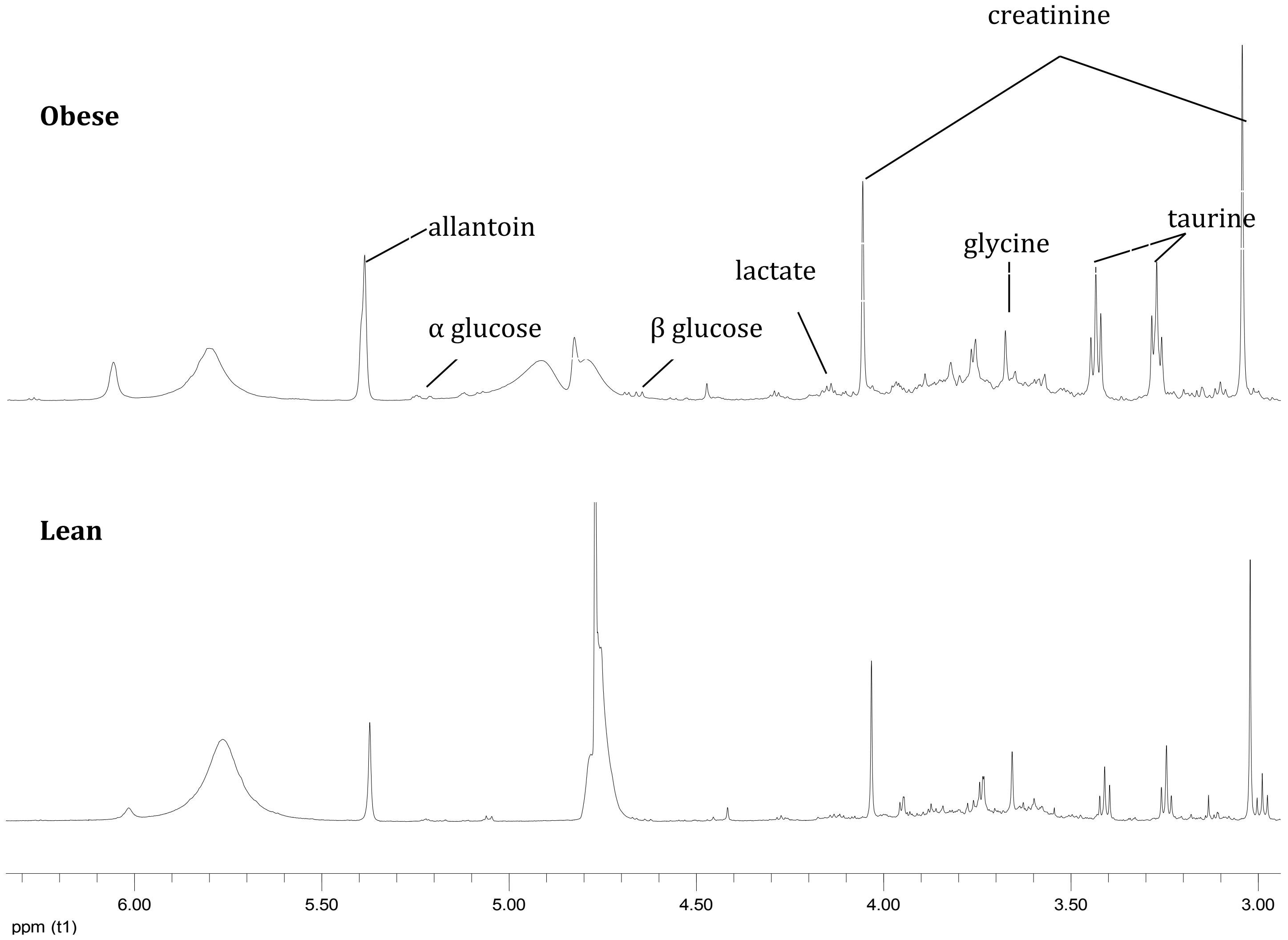

Obese
Lean
creatinine
allantoin
taurine
glycine
lactate
α glucose
β glucose
6.00
5.50
5.00
4.50
4.00
3.50
3.00
ppm (t1)

Typical 500 MHz ^{1}H NMR spectra of urine Typical 500 MHz ^{1}H NMR spectra of urine collected from a Sprague- Dawley rat fed a high fat diet (obese) and a Sprague- Dawley rat fed a normal diet (lean)) with expanded regions at δ 7.00 – 9.50, δ 1.00 – 2.50 and δ 3.00 – 6.00 ppm.
